# Development and validation of combined in vitro and in vivo assays for evaluating the efficacy of strontium-chelating compounds

**DOI:** 10.1038/s41598-025-06618-1

**Published:** 2025-11-28

**Authors:** Nóra Varga, Viola Pomozi, Eszter Kozák, Zsuzsa Erdei, Szilárd Tóth, Adriána Kutás, Zoltán Mucsi, Zsolt Rapi, Beatrix Kovács, Anett Matuscsák, Olivér Bánhídi, Csaba Váradi, Tamás Rácz, Katalin Német, Béla Viskolcz, Áron Szepesi

**Affiliations:** 1 Creative Cell Ltd., Budapest, 1119 Hungary; 2 Institute of Molecular Life Sciences, HUN-REN Research Centre for Natural Sciences, Budapest, 1117 Hungary; 3 Advanced Materials and Intelligent Technologies Higher Education and Industrial Cooperation Centre, University of Miskolc, Egyetemváros, C/2 épület 4.hajó dél I. 108., Miskolc, 3515 Hungary; 4 Department of Organic Chemistry and Technology, Faculty of Chemical Technology and Biotechnology, Budapest University of Technology and Economics, Műegyetem rkp. 3., Budapest, 1111 Hungary; 5 Laboratory of 3D Functional Network and Dendritic Imaging, HUN-REN Institute of Experimental Medicine, Szigony st 43., Budapest, 1083 Hungary; 6 Stratoxer(s) Ltd., Várpalota, 8100 Hungary

**Keywords:** Radioactive strontium, Strontium-chelating compounds, Bone differentiation, Bone incorporation prevention, Cell biology, Environmental sciences, Nuclear chemistry, Mesenchymal stem cells

## Abstract

The aim of this study is to develop a combined in vitro and in vivo assay for the comprehensive efficacy evaluation of strontium-chelating compounds. The complexation and removal of harmful radioactive strontium (^90^Sr) from the living organism may be necessary in the event of an accidental nuclear disaster or nearby nuclear war event, to prevent its incorporation into bones and thus avoid its long-term harmful health effects. To validate the model, we used a strontium-selective chelator macrocyclic substance (Decorporol) in our measurements, which potentially could be approved as a drug without significant toxic side effects. It has been proven to effectively remove strontium before it is incorporated into bones without significantly affecting calcium homeostasis in the body, and without relevant toxicity or side effects. In this study, we established an extremely sensitive test system that allows for the examination of chelator molecules in preventing the incorporation of non-radioactive SrCl_2_ into mineralized extracellular matrix in cell culture. We also optimized an in vivo mouse model suitable to investigate strontium incorporation and the effect of chelators. These assays also provide an opportunity for the safe evaluation of similar compounds.

**Supplementary Information:**

The online version contains supplementary material available at 10.1038/s41598-025-06618-1.

## Introduction

One of the isotopes of strontium is the radioactive strontium-90 (^90^Sr), which does not occur naturally in the environment but is released in significant quantities during nuclear incidents such as reactor accidents and atomic bomb detonations.

Among the radionuclide contaminations resulting from nuclear accidents (most commonly ^134^Cs, ^137^Cs, and ^90^Sr), ^90^Sr is of particular importance due to its long half-life, high water solubility, and strong affinity for bone tissue, where it accumulates and can replace calcium. While radioactive cesium isotopes (^134^Cs and ^137^Cs) also exhibit bioaccumulation, their distribution in soft tissues and faster biological half-life result in different toxicokinetics compared to strontium^1–5^. This represents one of the main long-term hazards of radioactive contamination: when inhaled or ingested with food via air or precipitation, strontium behaves similarly to calcium ions during the natural mineralization processes of living organisms and is efficiently incorporated into bone tissue, from where subsequent removal is not possible^6,7^. The incorporated ^90^Sr remains in the bone for a long time: the isotope has a half-life of 28.9 years, and the estimated elimination rate is only 7.5% of the total amount per year^8^. Radiation from the decay of ^90^Sr in bones - along with other radionuclides - can disrupt hematopoiesis and immune function in the bone marrow, significantly increase the frequency of malignant lesions in bone tissue and impair bone physiology and DNA repair mechanisms in bone and its surroundings^9–11^.

Selective removal of ^90^Sr incorporated into bone tissue is not possible, so the only way to avoid these serious consequences is to prevent the isotope from being incorporated. The aim of the planned project is to develop in vitro and in vivo model systems suitable for studying the effects of strontium exposure and testing chelator compounds. These chelators could assist in the rapid elimination of strontium from the body, thereby preventing or reducing its irreversible incorporation into bones.

Chelators such as dimercaprol, succimer, D-penicillamine, or DTPA (diethylentriamene pentaacetate), which are employed in the treatment of various metal poisonings (e.g., mercury, lead, chromium, cadmium), are capable of binding different metal ions when administered to living organisms^12^. However, the use of chelators has been limited in certain cases due to significant adverse effects; for instance, some agents have demonstrated toxicity, or the complexes they form with target metals may induce oxidative stress, thereby inflicting cellular and tissue damage (e.g., EDTA)^13,14^. In contrast, compounds such as DMSA (meso-2,3-dimercaptosuccinic acid) and DMPS (2,3-dimercapto-propanesulphonate) continue to be employed in heavy metal detoxification protocols, largely due to their relatively low toxicity profiles^15^.

Currently, there are few reliable and selective methods available for the removal of strontium. A study from 1959 indicated that treatment with ammonium chloride could enhance the urinary excretion of strontium isotopes^16^. However, as these data were obtained from human volunteer studies, direct evidence regarding the extent of bone incorporation was not available, thereby complicating the assessment of this treatment’s effectiveness in preventing strontium accumulation in bone tissue.

For the management of strontium contamination, current protocols adhere to the recommendations of the International Atomic Energy Agency (IAEA) by employing calcium gluconate therapy. Calcium, being a non-radioactive element, competes with the strontium radionuclide for uptake sites, effectively displacing radiostrontium from sites of bone deposition. Nevertheless, the efficacy of this therapeutic approach has been questioned, and its selectivity for strontium has yet to be definitively established^17^.

In this study, we evaluated Decorporol (Chemical Formula: C₁₈H₂₆CaN₂Na₂O₁₂; MW: 548.47) [calcium-disodium-2,2’-(1,4,10,13-tetraoxa-7,16-diazacyclooctadecane-7,16-diyl)dimalonate], a synthetic chelator developed for radiostrontium decorporation^18^. Based on historical data indicating preferential binding to Sr²⁺ over Ca²⁺, Decorporol was selected as a reference compound in our in vitro and in vivo experiments to assess its efficacy in reducing the incorporation of radiostrontium into bone tissue.

To quantify the effectiveness of Decorporol in reducing strontium incorporation into tissues, we utilized Inductively Coupled Plasma Atomic Emission Spectroscopy (ICP-AES). In analytical chemistry, the ICP-AES method generates high-temperature plasma (typically with argon gas) to atomize, ionize samples, whose resulting ions are then analyzed using mass spectrometry or optical emission spectroscopy to determine elemental composition^19,20^. This technique allowed us to accurately measure the concentration of strontium in various biological samples post-treatment. The sensitivity and precision of ICP-AES were crucial in demonstrating that Decorporol significantly decreases strontium levels in bone tissue, confirming its potential as a therapeutic agent for strontium decorporation. To avoid radioactive contamination in the planned experiments, we used stable, non-radioactive SrCl_2_ solution instead of ^90^Sr isotope during treatments. This approach is advantageous due to its non-radioactive and non-toxic nature, and the planned sensitive ICP-AES supplemented with UPLC-MS (ultra-performance liquid chromatography-mass spectrometry) measurement-based tests, is suitable for the precise determination of strontium quantities in cell cultures, as well as in blood, urine, feces, and bone samples collected after chelator treatments.

Our advanced in vitro and in vivo model systems, incorporating modern technologies such as ICP-AES and UPLC-MS, provide a valuable platform for the identification and characterization of novel strontium-chelating compounds. These approaches may support the future development of similarly acting agents. Moreover, the use of in vitro cell-based pre-screening allows for the early assessment of compound efficacy, contributing to a more ethical and efficient research process by reducing the reliance on animal models.

## Results

### In vitro human mesenchymal stem cell models for detection of strontium incorporation during osteogenic differentiation

#### Model description

**Fig. 1:**
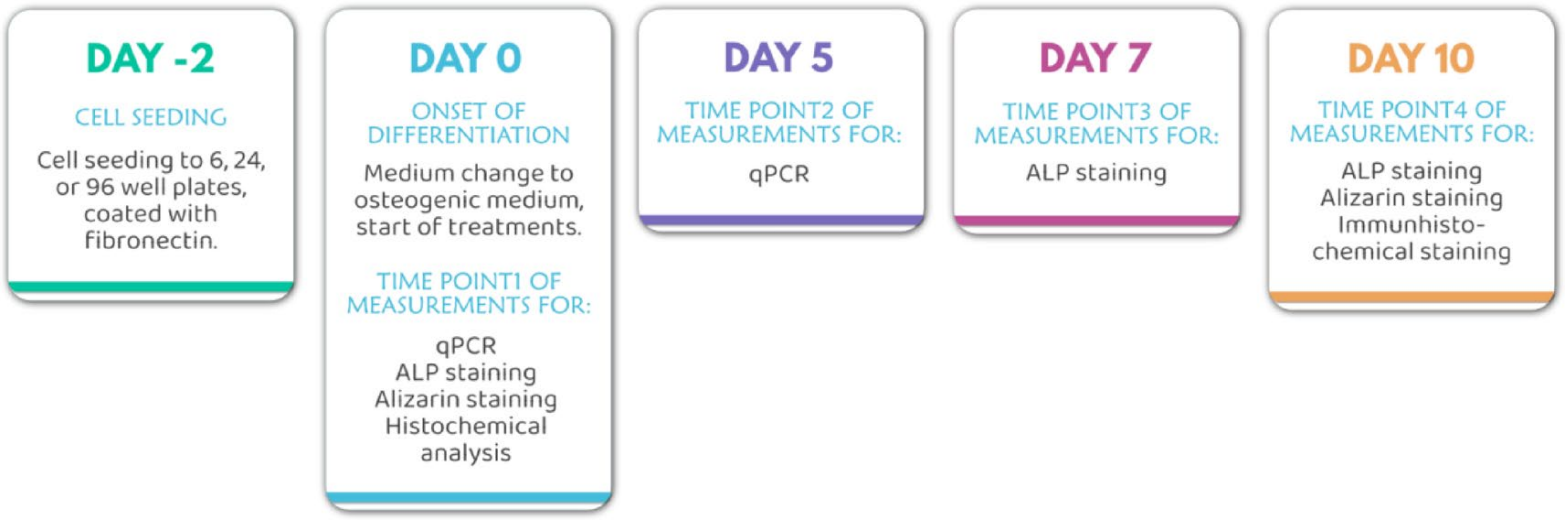
Schematic representation of the in vitro bone differentiation model of mesenchymal stem cells.

From the literature, among the known osteogenic differentiation techniques^21,22^, we applied the technique previously described and validated by our group^23^ to investigate the osteogenic potential of bone marrow-derived MSC (BM-MSCs) and adipose-derived MSC cells (Ad-MSCs). Using this technique, the directional differentiation of MSCs towards bone can be reliably performed. The schematic diagram illustrating the differentiation process used by us is shown in Fig. 1.

### Optimization of the osteogenic differentiation model for detection of strontium using ICP-AES technique

In the first step, we optimized the in vitro differentiation model by differentiating cells on six-well culture plates. This size of area provided the most reliable differentiation and sample collection for ICP-AES measurements.

Since alkaline phosphatase (ALP) is one of the most important markers of bone differentiation^24^, we conducted a kinetic analysis of ALP mRNA expression and enzyme activity on days 0, 5, and 7 of differentiation. The results showed optimal ALP detection on the 7th day, at both mRNA and protein levels. Therefore, in subsequent experiments, we focused on detecting ALP expression on the 7th day (Supplementary Fig. 1).

After optimizing the osteogenic differentiation protocols, we investigated the strontium incorporation during in vitro differentiation. In these experiments, mesenchymal stem cells were kept in osteogenic medium supplemented with various concentrations of SrCl_2_ (0.5 mM, 1 mM, 2 mM). Subsequently, on the 10th day of differentiation, the amount of incorporated strontium into the bone/osteo cells was examined using the ICP-AES technique. These results demonstrated that strontium at different concentrations could be reliably detected in the extracellular matrix of the bone-forming cells in vitro (see Supplementary Fig. 2).

### Prevention of the SrCl_2_ incorporation during osteogenic differentiation of human mesenchymal stem cells using chelator decorporol

**Fig. 2:**
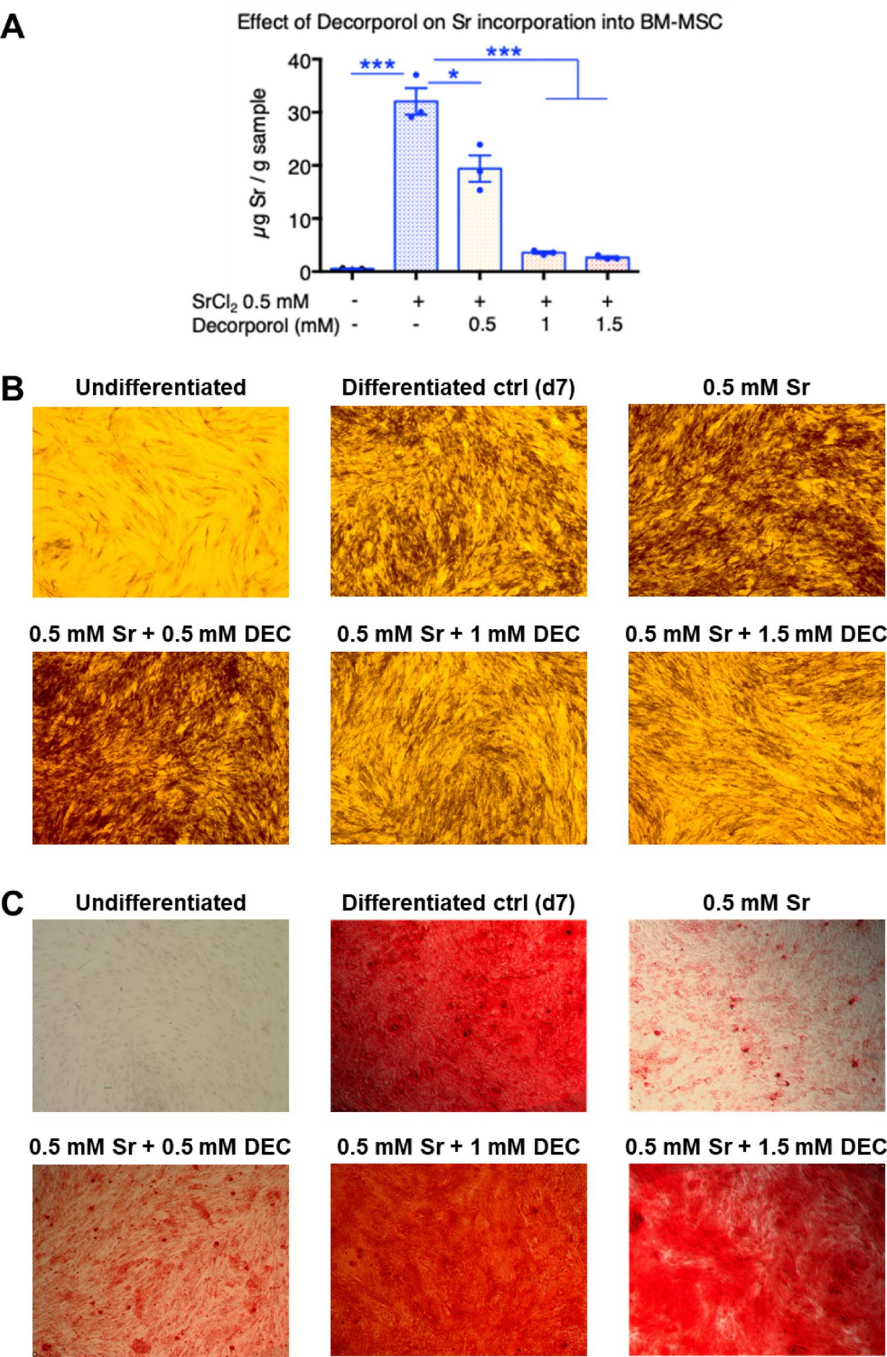

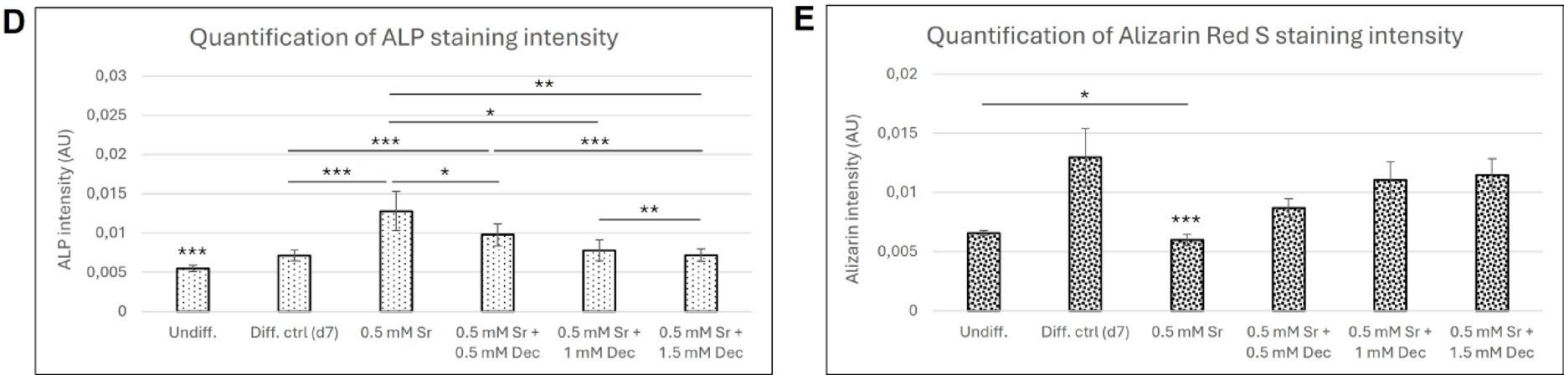
The bone marrow derived mesenchymal stem cells were treated with 0.5 mM strontium (Sr) during the differentiation process, and decorporol (DEC) was added in parallel with the strontium in ratios of 1:1, 1:2, and 1:3 (0.5 1, 1.5 mM respectively). Detection of strontium incorporation by ICP-AES technique on the 10th day of osteogenic differentiation of bone marrow-derived MSCs (BM-MSC). The Y-axis represents the absolute amount of incorporated strontium in micrograms [µg Sr] (**A**). Detection of the osteogenic marker ALP protein activity using ALP staining on the 7th day of osteogenic differentiation (**B**). Alizarin Red S staining demonstrating calcium deposition (red) of the matured bone matrix after 10 days of osteogenic induction. Images captured at 4x magnification under a light microscope (**C**). Quantification of ALP staining intensity. RGB-decomposed images were analyzed in MATLAB. SrCl₂ significantly increased ALP expression, which was reversed by decorporol (**D**). Quantification of Alizarin red S staining intensity. Image analysis revealed decreased calcium deposition in SrCl_2_-treated cells, restored by decorporol co-treatment (**E**). Statistical significance is indicated as *p* < 0.05 (*), *p* < 0.01 (**), and *p* < 0.001 (***).

Based on previous data regarding Decorporol, we investigated whether Decorporol prevents strontium incorporation into bone cells in our in vitro test system. In these experiments, primary mesenchymal stem cells (MSCs) derived from bone marrow (BM-MSC) and adipose tissue (Ad-MSC) were differentiated towards the osteogenic lineage using the protocol described previously. The differentiating cells were treated with 0.5 mM strontium alone and in parallel with Decorporol at various concentrations. The results clearly demonstrate that compared to untreated cells, Decorporol applied in 0.5, 1 or 1.5 mM alongside 0.5 mM strontium effectively prevented strontium incorporation into the bone cells in case of BM-MSC (see Fig. 2A). We confirmed these results using a different cell type: MSCs derived from adipose tissue (Ad-MSC) were also differentiated towards the osteogenic lineage using the same protocol. Even though the adipose-derived MSCs showed lower differentiation capacity compared to bone marrow-derived MSCs, the effect of Decorporol preventing strontium incorporation into the cells was obvious (Supplementary Fig. 3A), as demonstrated by ICP-AES technique.

The strontium and Decorporol treatments did not affect osteogenic differentiation, as confirmed by alkaline phosphatase (ALP) staining, a marker for osteogenic differentiation, and Alizarin Red S staining, which detects calcium deposition both in BM-MSC (see Fig. 2B–E) and Ad-MSC (Supplementary Fig. 3B).

**Fig. 3:**
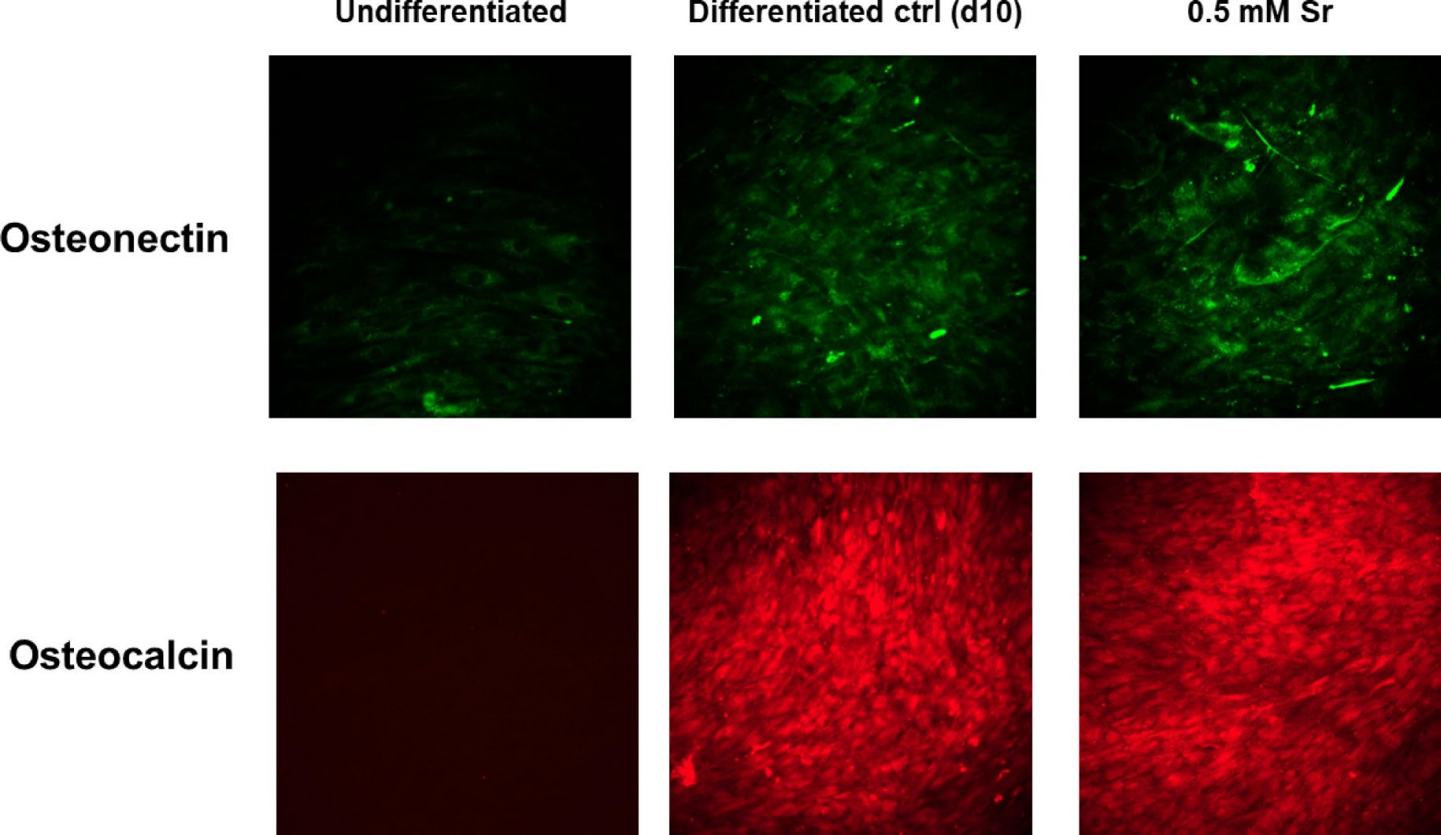
Immunocytochemical staining of the osteogenic marker osteonectin (green) osteocalcin (red) on the 10th day of differentiation in control and strontium-treated BM-MSC samples.

In our Alizarin Red S staining results, we observed that samples treated with 0.5 mM strontium showed strontium incorporation into bone cells, as revealed by ICP-AES analysis. Moreover, ALP expression results indicated that strontium enhanced early markers of osteogenic differentiation (Fig. 2B, D), although this was not reflected in robust mineral deposition as shown by Alizarin Red S staining (Fig. 2C, E). However, we noted weak staining with Alizarin Red S in samples treated only with strontium on the 10th day of differentiation. To confirm whether strontium-treated cells indeed differentiated into bone cells, we simultaneously examined the expression of two osteogenic markers using immunocytochemical staining on the 10th day of differentiation of BM-MSC samples (Fig. 3). Our results confirmed that these cells are indeed differentiated into bone lineage despite the weak Alizarin Red S staining.

To confirm the in vitro results, we aimed to employ an in vivo model system to measure strontium incorporation into bone tissue and assess the protective capacity of Decorporol. Therefore, our aim was to establish a mouse model by determining the optimal experimental setup, including dosage, mode of delivery, and duration of treatment for both strontium and Decorporol. We then sought to evaluate whether Decorporol can prevent strontium incorporation into bone tissue in vivo.

### Strontium and decorporol absorption in mice

**Fig. 4:**
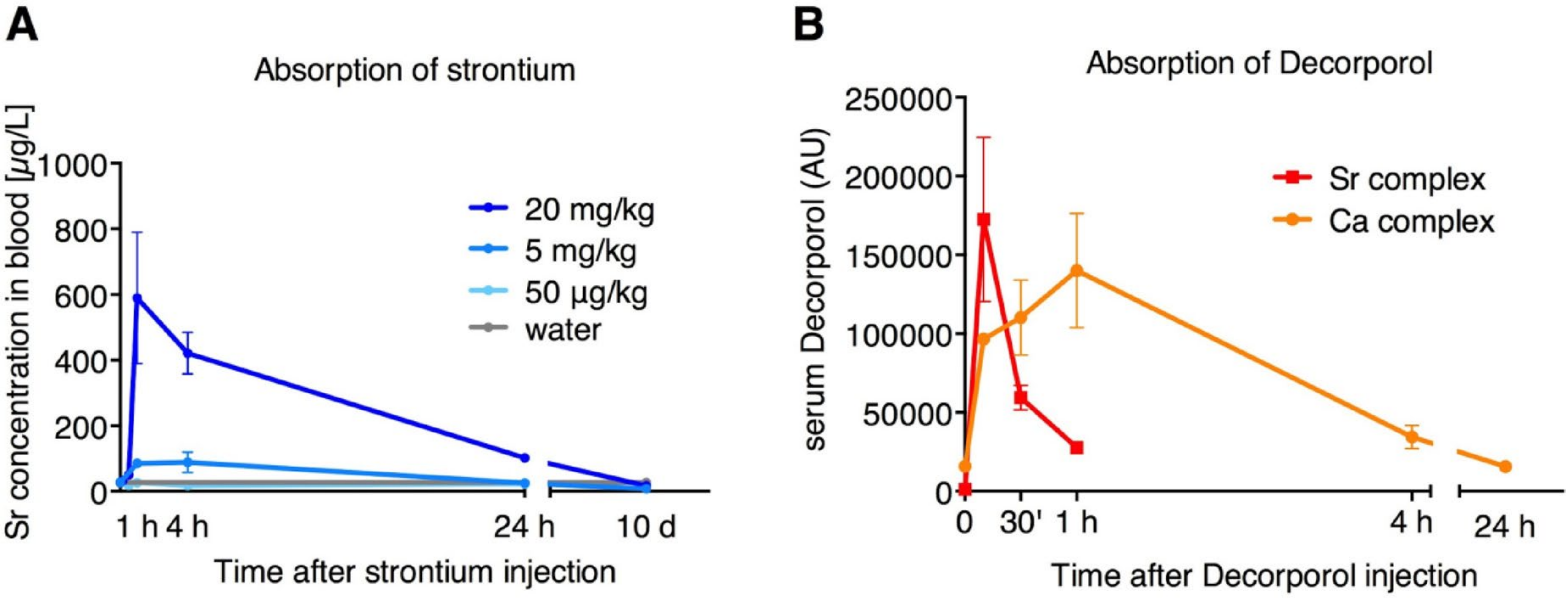
Absorption kinetics of strontium and Decorporol in mice: Strontium (Sr) was administered by oral gavage and detected using the ICP-AES technique. Strontium was absorbed into the circulation in a dose-dependent manner and was cleared within 24 h (**A**). Mice were administered 40 µmol/kg Decorporol via intraperitoneal injection 30 min after strontium gavage. Decorporol was detected in the blood of mice using the UPLC-MS technique. Strontium-bound Decorporol appeared in the circulation earlier than the calcium-bound form, confirming a higher affinity of Decorporol for strontium (**B**). (*n* = 3)

First, the absorption of strontium into the circulation of mice was tested using three different doses: 50 µg/kg, 5 mg/kg, and 20 mg/kg. Strontium was administered via oral gavage (modeling oral consumption of strontium). Blood samples were collected at different time points, and the strontium concentration was determined using ICP-AES technique. Our results indicate that strontium was absorbed into the blood within one hour in a dose-dependent manner (Fig. 4A). Urine and feces were also collected from the mice after strontium administration, and strontium content was measured. The results show that strontium was completely cleared from the circulation after 24 h (Supplementary Fig. 4A, B).

Decorporol was administered to mice intraperitoneally at a dose of 40 µmol/kg, 30 min after strontium administration. Using MS technique, Decorporol can be detected in the blood of mice, and the calcium- and strontium-bound forms can be distinguished. Our results show that strontium-bound Decorporol appeared in the circulation earlier than the calcium-bound form (Fig. 4B), indicating a higher affinity of Decorporol for strontium, as expected. Based on data from the WO 91/10,655 patent, the Sr^2+^/Ca^2+^ ratio for Decorporol is 1.2. Decorporol was cleared from circulation within four hours.

### Strontium incorporation into bone tissue, the effect of decorporol

**Fig. 5:**
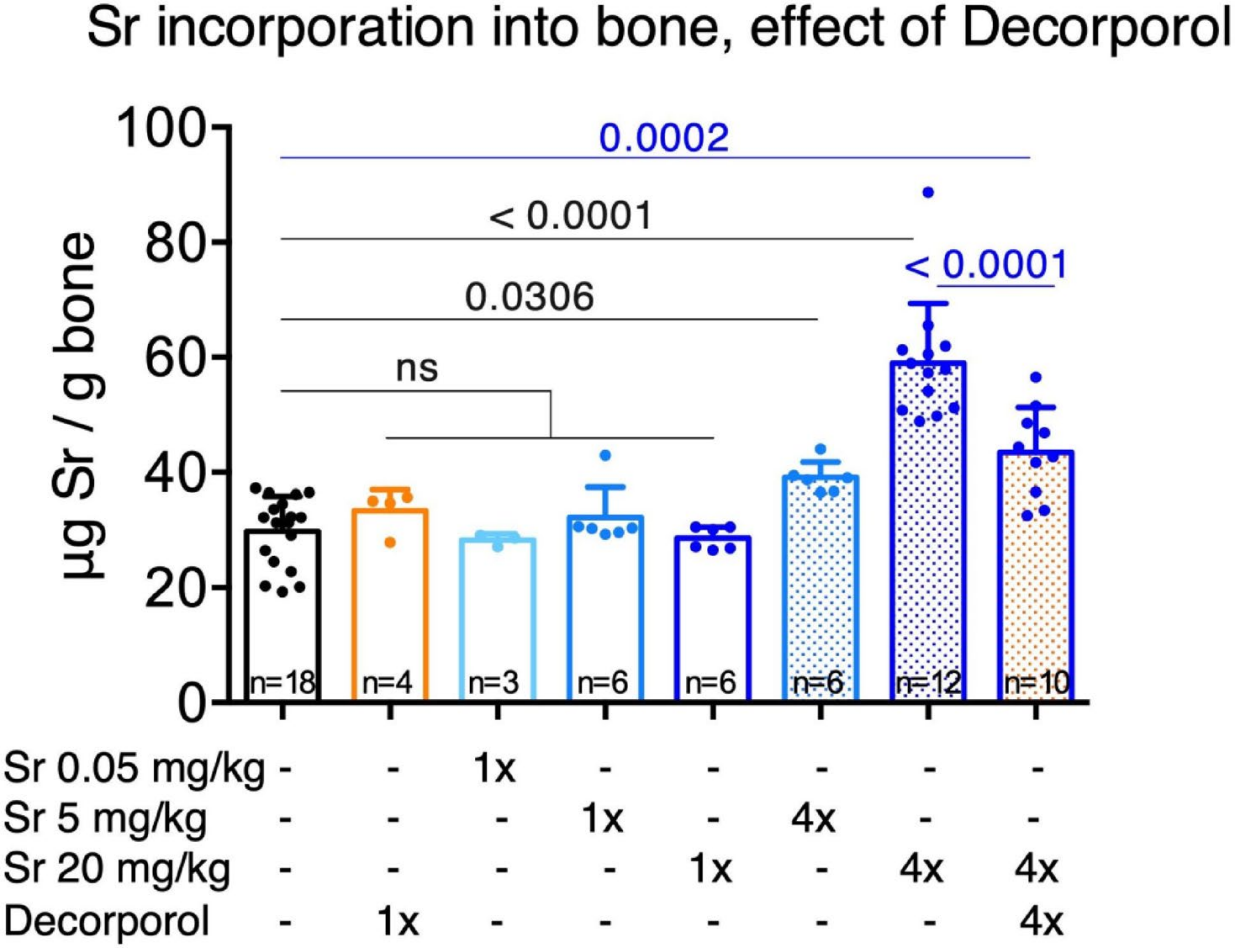
Strontium (Sr) did not integrate into bone tissue after a single dose (solid blue bars), but daily doses of 5 or 20 mg/kg for 4 days led to significant integration (dotted blue bars). Decorporol treatment effectively prevented Sr incorporation into bone (blue bar with orange dots). Data are shown as the mean ± SD with individual biological replicates indicated by dots. Animal numbers are also indicated at the bottom of each bar. Significance was measured by one-way ANOVA with Dunnett’s test of multiple comparisons relative to untreated controls (black numbers) or relative to 4× Sr combined with 4x Decorporol treatment (blue numbers).

One single dose of either 0.05, 5 or 20 mg/kg did not result in significant strontium incorporation into bone tissue. However, administration of strontium once a day for 4 days resulted in ~ 34% and ~ 88% increase in femur strontium in the case of 5 mg/kg and 20 mg/kg of strontium intake, respectively (Fig. 5).

For further experiments to investigate strontium incorporation into bone tissue, the 20 mg/kg daily dose for 4 days protocol was chosen. When Decorporol treatment was given 30 min after mice were exposed to strontium, Decorporol could decrease the amount of strontium incorporated in bone tissue by ~ 26% after 4 days (compared to strontium treated mice without Decorporol administration) (Fig. 5).

## Discussion

Over the past 80 years, developments in nuclear energy have expanded significantly. Despite its clear advantages, its use carries considerable risks, even when the highest safety standards are applied. Radioactive isotopes such as iodine (¹³¹I and ¹²⁹I), cesium (^134^Cs and ^137^Cs), and strontium (^90^Sr), typically released during nuclear fission events such as atomic explosions or nuclear power plant accidents, pose serious health hazards. These isotopes are also considered potential threats in hypothetical nuclear terrorism scenarios involving radiological dispersal devices (RDDs)^25,26^.

There is already an established drug treatment for displacing radioactive iodine using iodine-containing tablets^27^. Although certain therapeutic strategies - such as calcium gluconate treatment recommended by the International Atomic Energy Agency (IAEA) - have been employed to mitigate radiostrontium contamination, their efficacy and selectivity remain uncertain^17^. Therefore, there is a pressing need to develop experimental radioprotective agents into clinically applicable pharmaceuticals.

Radioactive strontium can enter the body through inhalation or ingestion of contaminated food and water^28,29^. While strontium shares certain chemical similarities with calcium and can be incorporated into bone tissue, recent biokinetic models indicate that strontium exhibits distinct distribution patterns within the body, particularly in relation to red bone marrow and adipose tissue. These differences are important for accurately assessing radiotoxic effects and long-term health risks.

Radioactive strontium can enter the body of exposed personnel through inhalation^28^ or ingestion of contaminated food or drinking water^29^. Strontium is located below calcium in the periodic table and shares several physicochemical properties with it, including the ability to incorporate into the bone mineral matrix in place of calcium. Due to this similarity, strontium partially follows the biokinetic pathway of calcium, particularly in bone tissue. However, recent biokinetic models - such as those described in ICRP Publication 134 - highlight that strontium exhibits distinct distribution and retention characteristics in other tissues, including red bone marrow and adipose tissue^30^. Once integrated into bone, radioactive ⁹⁰Sr poses a significant radiotoxic risk due to its long half-life of 29 years, contributing to cumulative radiation damage and increasing the risk of malignant transformations in bone, bone marrow, and surrounding soft tissues^31^.

Previous researches^32,33^ have already demonstrated through in vivo animal experiments that some chelate-forming molecules can remove radioactive strontium ions from the living organism before they would be incorporated into bones. The challenge of this approach is creating chelating compounds that can selectively target strontium so they can more effectively facilitate its excretion. To remove strontium, chelate-type macrocyclic substances can be used, which form complexes that sequester strontium from the body and facilitate urinary excretion. Previously used open-chain complexing agents (e.g., EDTA and its relatives) are non-specific/non-selective for strontium and also remove essential metals needed by the body (e.g., calcium, magnesium, copper, manganese, iron). This may cause serious deleterious health effects by depleting these essential ions in the body^34^. On the other hand, this lack of selectivity also has a negative impact on the efficiency of any such treatment, since these other ions compete with ^90^Sr when binding occurs. In contrast, the macrocyclic compound previously studied has molecular structural parameters optimized for strontium^35^.

In this study, our goal was to develop and optimize both in vitro and in vivo models to measure strontium incorporation into bone and to evaluate the efficacy of chelators in selectively removing strontium and preventing its accumulation in cells and tissues. Given that strontium exposure affects the entire organism, in vivo models are essential. However, due to ethical and cost considerations, it is advisable to first test these chelator compounds on cell lines. We developed a sensitive in vitro system to study the effect of SrCl_2_ on the osteogenic differentiation of mesenchymal cells. Mesenchymal stem cells are ideal for investigating these strontium-selective chelators due to their healthy state, proliferative ability, and capacity to differentiate into bone tissue.

In line with previous literature data, we observed that the presence of SrCl_2_ accelerated mesenchymal stem cell (MSC) differentiation toward the osteogenic lineage, as evidenced by increased alkaline phosphatase (ALP) expression^36,37^. However, co-administration of Decorporol with SrCl_2_ (1:2 or 1:3 ratio) inhibited this effect, confirming the chelator’s ability to counteract strontium’s stimulatory impact on differentiation. Additionally, in the presence of 0.5 mM SrCl_2_, calcium deposition was reduced, as shown by Alizarin red staining. In contrast, Decorporol restored calcium accumulation to control levels, demonstrating its effectiveness in chelating strontium and mitigating its inhibitory effects on mineralization and differentiation.

Despite the well-documented variability in differentiation potential of mesenchymal stem cells (MSCs) based on tissue origin and donor background, we demonstrated the decorporation efficacy of Decorporol using one bone marrow-derived MSC (BM-MSC) line and one adipose-derived MSC (AD-MSC) line, each from a different human donor. While this focused cellular approach reflects practical constraints commonly encountered in translational research, the consistency of our findings across both MSC types suggests that the observed effect is not cell line-specific. These in vitro observations were further supported by our in vivo results obtained from a cohort of 59 animals, where Decorporol similarly reduced strontium accumulation. The high concordance between the in vitro and in vivo data highlights the robustness and translational relevance of our conclusions.

The use of the ICP-AES technique for the analysis of blood and bone samples in mice is a previously established method^38^. In this study, we demonstrate that a strontium-selective chelator can prevent the majority of strontium incorporation into the mineralized bone matrix, where strontium is predominantly deposited. To assess the efficacy of such a chelator, we employed Decorporol, a synthetic chelating agent developed in the 1980s for strontium decorporation^18^. While its physicochemical properties have not been fully characterized in peer-reviewed literature, the compound’s selective binding to Sr²⁺ over Ca²⁺ has been documented.

Our experimental setup, which included Decorporol, proved useful for testing additional chelating agents. We optimized an in vivo mouse model to quantify strontium accumulation in bone tissue, using relatively high doses of both strontium and Decorporol to achieve reproducible and measurable strontium incorporation into bones. These doses were significantly below the LD50 values (1500 mg/kg for SrCl_2_ in mice^39^ and > 2.5 mmol/kg for Decorporol in mice^18^). In addition to these in vivo toxicity thresholds, we also conducted in vitro cytotoxicity assays on various cell types—including MDCKII, HepG2, and primary human mesenchymal stem cells (MSCs)—to further assess the safety profile of both SrCl₂ and Decorporol. None of the tested substances induced cytotoxicity up to concentrations of 2 mM, and even the combined application of SrCl₂ and Decorporol at these high concentrations did not result in significant cell death (data not shown). These findings collectively suggest that neither the free chelator nor the Sr-chelator complex exhibits cellular toxicity within the biologically relevant concentration range.

When compared to currently used metal chelators, Decorporol appears to offer a favorable safety profile. Traditional agents such as EDTA, although effective, have been reported to induce oxidative stress and tissue damage due to their broad metal-binding properties and lack of selectivity^13,14^. More advanced compounds like DMSA and DMPS are still used in heavy metal detoxification because of their relatively lower toxicity^15^, yet these also lack specificity for alkaline earth metals such as strontium. Furthermore, the clinically used calcium gluconate, commonly administered to mitigate strontium exposure^12^, does not act through selective chelation, and its efficacy is primarily based on ionic competition rather than direct binding, limiting its application for strontium-specific interventions.

In contrast, Decorporol is a structurally optimized, high-affinity chelator for divalent cations, and our findings suggest that it may exhibit higher selectivity for strontium ions, while remaining non-toxic under both in vitro and in vivo conditions. Although comprehensive toxicological and pharmacokinetic profiling will be required in future studies, our current work provides a proof-of-concept for the development of a targeted and biocompatible chelation-based strategy. The aim of the present study was not to establish a therapeutic application, but rather to develop and validate a test system that can model the biological behavior of strontium and evaluate the efficacy of candidate chelators such as Decorporol in a controlled setting.

Both single doses and a cumulative dosing regimen − 1 daily dose of SrCl_2_ and Decorporol each for 4 consecutive days - were tested. The 4× daily dosing resulted in a significant elevation of bone Sr content, in the context of which the effect of Decorporol treatment could be reliably assessed. The high sensitivity of the ICP-AES technique eliminated the need to use radioactive strontium.

In the experimental setup SrCl_2_ was administered orally, and for the chelator a different route of administration was chosen to prevent the pre-formation of Decorporol-Sr complex. Decorporol was given intraperitoneally to the mice half an hour after strontium load. Based on the results obtained under the specific experimental conditions tested, Decorporol treatment reduced strontium incorporation into bone tissue without apparent adverse effects. Future studies are warranted to define the therapeutic time window after strontium exposure during which chelator treatment remains effective. While further investigations are required to assess dosing strategies, potential alternative routes of administration, and long-term safety - including effects on calcium homeostasis - our in vitro and in vivo models provide a valuable preclinical platform for the evaluation of candidate chelating agents.

To the best of our knowledge, no one has previously examined the effectiveness of such chelator molecules simultaneously on an in vitro healthy cell line and an in vivo animal model.e

## Materials and methods

A comprehensive list of all reagents, antibodies, and materials employed in this study, along with their suppliers and catalogue numbers, is provided in Table 1 for reference.

**Table 1:** Key resources.

Reagent or resource	Source	Identifier
Antibodies		
Anti-hSPARC mouse monoclonal IgG	R&D Systems	Cat# MAB941
Rabbit polyclonal osteocalcin antibody	Abcam	Cat# ab93876
Alexa-fluor 488 donkey anti-mouse IgG (H + L)	Life Technologies	Cat# A21202
Alexafluor 568 goat anti-mouse IgG (H + L)	Invitrogen	Cat# A11004
DAPI (4′,6-diaminido-2-phenylindole dihydrochloride)	Sigma-Aldrich	Cat# D8417-1MG
Chemicals, peptides and recombinant proteins		
DMEM/F-12 (1:1) (1x) + GlutaMAX-I	Thermo Fisher Scientific	31331-028
Fetal bovine serum (FBS)	Thermo Fisher Scientific	A5256701
l-glutamine	Thermo Fisher Scientific	25030-024
Gentamicin	Thermo Fisher Scientific	15750-045
Human FGF - basic	Peprotech	#100-18B-100 UG
Hu plasma fibronectin	Millipore	FC010
Knockout-DMEM	Thermo Fisher Scientific	10829-018
MEM NEAA	Thermo Fisher Scientific	11140-035
2-mercaptoethanol	Thermo Fisher Scientific	31350-010
Beta-glycerophosphate disodium salt hydrate	Sigma-Aldrich	G9422-504
2-phospho-l-ascorbic acid trisodium salt	Sigma-Aldrich	49752-10G
Dexamethasone	Sigma-Aldrich	D1756-100MG
Paraformaldehyde (PFA)	Fluka	76,240
Alizarin-red S stain	Sigma-Aldrich	A5533-25G
BCIP/NBT liquid substrate system	Sigma-Aldrich	SLCN3462
Strontium chloride hexahydrate	Sigma-Aldrich	255,521
PureLink™ RNA mini kit	Thermo Fisher Scientific	12,183,025
High-capacity cDNA reverse transcription kit	Thermo Fisher Scientific	4,368,814
TaqMan™ universal PCR master mix	Thermo Fisher Scientific	4,364,338
GAPDH TaqMan™ gene expression assay	Thermo Fisher Scientific	Hs99999905_m1
ALP TaqMan™ gene expression assay	Thermo Fisher Scientific	Hs01029144_m1

### Synthesis of decorporol

**Fig. 6:**
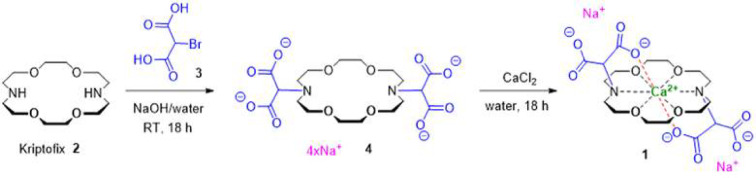
Synthetic route of decorporol (**1**).

The current synthesis of Decorporol (**1**) is based on the original synthesis^40^, published in patent WO 91/10,655 (Fig. 6). In the first step, the alkylation of 1,4,10,13-tetraoxa- 7,16-diazacyclooctadecane (Kriptofix, **2**) is carried out using 2-bromomalonic acid (**3**) in aqueous solution of NaOH at 60–65 °C, obtaining the tetrasodium salt (**4**). Without isolation and after the cooling to RT, 1 equivalent of CaCl_2_ was added to the solution and the dinatrium mono calcium salt of the corona ether (Decorporol, **1**) was crystalized, then filtered off. The synthetic pathway of Decorporol is illustrated in Fig. 6.

### Isolation, culturing, differentiation and strontium/decorporol treatment of cells

#### Isolation and culturing of MSCs

All samples utilized in this study were obtained from consenting healthy donors, following approval by the Ethical Committee of the Hungarian Medical Research Council (ETT; ID: 24083-3/2013/HER). All methods were carried out in accordance with relevant guidelines and regulations. All experimental protocols were approved by the Hungarian Medical Research Council, and informed consent was obtained from all donors or their legal guardians.

Mesenchymal stem cells (MSCs) derived from bone marrow (*n* = 1) and adipose tissue obtained via liposuction (*n* = 1) were isolated according to previously established protocols^41^. Cells were maintained in growth medium composed of DMEM/F-12 (1:1) (1x) + GlutaMAX-I (Thermo Fisher Scientific, Cat: 31331-028) supplemented with 10% fetal bovine serum (FBS) (Thermo Fisher Scientific, Cat: A5256701), 50 µg/mL gentamicin (Thermo Fisher Scientific, Cat: 15750-045), and 1 ng/mL human FGF - basic (Peprotech, Cat: #100-18B-100 UG). Cultures were allowed to reach approximately 80–90% confluency before subculturing with 0.25% trypsin for 3 min. MSCs used in the experiments presented here were between the 6th and 12th passages.

#### Osteogenic differentiation

For differentiation experiments, cells were seeded onto fibronectin-coated (Hu plasma fibronectin, Millipore, Cat: FC010) 6-well culture plates at a density of 140,000 cells/well for subsequent analysis via ICP-AES measurement, Alizarin Red S and alkaline phosphatase (ALP) staining. Following seeding, cells were allowed to differentiate for 10 days with medium changes every 2 days. Osteogenic differentiation of MSCs was induced using differentiation medium composed of the following: 250 ml Knockout-DMEM (Thermo Fisher Scientific, Cat: 10829-018), 25 ml (10%) FBS (Thermo Fisher Scientific, Cat: A5256701), 2.5 ml l-glutamine (Thermo Fisher Scientific, Cat: 25030-024), 2.5 ml MEM NEAA (Thermo Fisher Scientific, Cat: 11140-035), 0.25 ml 2-mercaptoethanol (Thermo Fisher Scientific, Cat: 31350-010), 0.25 ml Gentamicin (Thermo Fisher Scientific, Cat: 15750-045), 540 mg beta-glycerophosphate disodium salt hydrate (Sigma-Aldrich, Cat: G9422-504), 12.5 mg 2-phospho-L-ascorbic acid trisodium salt (Sigma-Aldrich, Cat: 49752-10G), and 100 nM Dexamethasone (Sigma-Aldrich, Cat: D1756-100MG).

#### Strontium and decorporol treatment

Strontium and Decorporol treatments were initiated on the first day of differentiation by adding these materials to the osteogenic differentiation medium at a final concentration of 0.5 mM SrCl_2_ (Strontium chloride hexahydrate, Sigma-Aldrich, Cat: 255521), either alone or in combination with 0.5, 1, or 1.5 mM Decorporol. SrCl₂ treatments were performed using freshly prepared solutions from a 200 mM aqueous stock, which were renewed every other day during medium changes. The treatment continued until the completion of the differentiation process.

In all experiments cell culture samples were used in three parallels.

### Sample preparation for ICP-AES measurements

We aimed to investigate the incorporation of strontium during in vitro mesenchymal stem cell osteogenic differentiation using the ICP-AES technique. For ICP-AES measurements, samples were washed twice with distilled water, and cells were subsequently detached from the culture plates using a cell scraper. The collected cells were then centrifuged in Eppendorf tubes for 5 min. After removing the supernatant, the cell pellets were treated with 300 µl of 65% nitric acid. The tubes were placed in a fume hood for 1 h with ventilation and sealed afterward.

### Alizarin red S staining

For Alizarin Red S staining, cells were fixed with 4% Paraformaldehyde (PFA, Fluka, Cat: 76240) solution for 15 min, followed by washing twice with PBS solution and three washes with distilled water. Subsequently, cells were incubated with Alizarin Red S stain (Sigma-Aldrich, Cat: A5533-25G) for 1 h, followed by three gentle washes with distilled water.

### Alkaline phosphatase activity

For alkaline phosphatase (ALP) enzyme activity detection, cells were fixed with 4% PFA solution, washed three times with PBS, and then incubated with ALP reagent (BCIP/NBT Liquid Substrate System, Sigma-Aldrich, Cat: SLCN3462) for 1 h. After three washes with PBS, images were captured using a light microscope as described previously^42^.

### Immunocytochemistry

Cells were fixed with 4% PFA for 15 minutes and incubated with unconjugated primary antibodies against bone differentiation markers for 1 hour at room temperature. The antibodies were diluted according to the manufacturer’s instructions: osteonectin (anti-hSPARC Purified Mouse Monoclonal IgG, R&D Systems, Cat: MAB941) and osteocalcin (Rabbit Polyclonal Osteocalcin antibody, Abcam, Cat: ab93876). For fluorescent detection, the following secondary antibodies were used: Alexa Fluor 488 (for osteonectin staining - Life Technologies, Alexa Fluor 488 donkey anti-mouse IgG (H + L), Cat: A21202) and Alexa Fluor 568 (for osteocalcin staining - Invitrogen, Alexa Fluor 568 goat anti-rabbit IgG (H + L), Cat: A11004). Nuclei were counterstained with DAPI (Sigma, 4’,6-diamidino-2-phenylindole dihydrochloride, Cat: D8417).

### Immunocytochemistry

Images of osteonectin, osteocalcin, alizarin, and alkaline phosphatase stainings were analyzed in MATLAB (MathWorks) using custom scripts. JPEG images were decomposed into their red, green, and blue channels, and mean intensity values were calculated separately for each color channel. These values were then averaged to obtain a general mean intensity for each image. The resulting data were subjected to statistical analysis using Student’s *t*-test, and group comparisons were visualized as bar charts.

### Gene expression analysis

Total RNA was isolated from undifferentiated and differentiated cells, followed by cDNA synthesis. The expression levels of the tissue-nonspecific alkaline phosphatase (ALP; TaqMan™ Gene Expression Assay ref num: Hs01029144_m1, Thermo Fisher Scientific) gene were quantified using TaqMan™ Universal PCR Master Mix reagent (Thermo Fisher Scientific, Cat: 4364338) with StepOne Plus qPCR instruments (Thermo Fisher Scientific). Gene expression levels were determined using the 2^−ΔCt^ method relative to glyceraldehyde-3-phosphate dehydrogenase (GAPDH; TaqMan™ Gene Expression Assay ref num: Hs99999905_m1, Thermo Fisher Scientific) gene expression as an internal reference.

### Animals

For the in vivo experiments 3 months old male BALB/c mice were used, purchased from the National Institute of Oncology, Department of Experimental Pharmacology (SPF Animal Facility) and housed at HUN-REN RCNS animal facility (permit no. PE/EA/00204-2/2023). Only male mice were used to reduce inter-individual variability and potential confounding effects of sex-related factors.

Mice were kept under routine laboratory conditions with a 12-hour light-dark cycle and with ad libitum access to water and chow. The HUN-REN RCNS Institutional Animal Care and Use Committee and the Hungarian national authority approved these studies (permit number: PE/EA/00712-6/2023) and experiments were conducted according to the national guidelines and the ARRIVE guidelines (Animal Research: Reporting of In Vivo Experiments).

### SrCl_2_ administration

SrCl_2_ was administered to mice via oral gavage technique. SrCl_2_ was dissolved in distilled water, and different doses were tested in our model (0.05 mg/kg, 5 mg/kg and 20 mg/kg) in a maximum volume of 150 µl. Mice got a single dose of SrCl_2_ or a daily dose for 4 consecutive days.

###  Decorporol administration

Decorporol was dissolved in 50 mM HEPES buffered physiological salt solution, and administered intraperitoneally. 40 µmol/kg Decorporol IP treatment was applied either alone, or 30 min after SrCl_2_ administration.

Both SrCl_2_ and Decorporol doses were below their LD_50_ concentrations^18,36^.

### Sample collection

#### Blood

For absorption kinetic studies, K_3_EDTA anticoagulated blood was collected from the tail vein of 3 mice per treatment group, and either subjected to acidic exploration with 1 ml of 65% HNO_3_ for ICP-AES analysis of SrCl_2_, or processed for Decorporol measurement. In the latter case, blood samples were centrifuged at 1000 g for 10 min, and plasma was stored at -80 °C until further processing. Before MS analysis, diluted plasma samples were filtered through 10 kDa PES membranes to exclude high MW plasma proteins.

The acidified samples for ICP-AES analysis contained 100 µl or less blood and were diluted to 10 ml.

#### Femur

To assess bone Sr incorporation, all animals were terminated 24 h after receiving their final SrCl_2_ dose. In the case of the single dose SrCl_2_ treatment, this occurred on the second experiment day, and in the 4x SrCl_2_ treatment group, this happened on day 5, 24 h after the last SrCl_2_ dose was administered. After the termination of mice, both femurs were removed and weighed (50–70 mg of mass), and each femur was dissolved in 1 ml 65% HNO_3_. and diluted to 100 ml. The strontium content of femur samples was determined by ICP-AES.

#### Urine and feces

To determine the amount of strontium excreted in the urine or feces, mice were placed in groups in metabolic cages after the administration of the compounds. During the 24 h spent in the cage, the supply of food and drinking water was continuously ensured. After 24 h, the volume of urine in the collection container and the weight of the feces were measured. For strontium measurement 25 µl urine was placed into 65% HNO_3_ and diluted to 10 ml. Feces were dried and approximately 0.15 g accurately weighted quantity was digested in 4 ml 65% nitric acid at 170 °C for 20 min in a microwave oven. After cooling to room temperature the samples were transferred to a volumetric flask and were filled up to 25 ml. In the course of the analysis further 10–100-fold dilution was performed depending on the Sr-content of the samples. The strontium content of the urine and feces samples was determined by ICP-AES method.

###  Strontium ICP-AES measurement

The determination of the strontium content of biological samples was performed by ICP-AES (Inductively Coupled Plasma Atom Emission Spectrometry). Samples stored in 65% HNO_3_ were diluted up to 10 ml or 100 ml with distilled water prior to analysis as it was described in the previous paragraph. The measurements were performed using the Varian Inc made 720 ES type simultaneous, multielement ICP spectrometer with axial view and nitrogen-purged Echelle optical system. The most sensitive ionic lines of strontium (407.771 and 421.552 nm) were used. Each sample was measured three times, with 10 s integration time for each. The calibration of the measurements was performed using a solution-series prepared from 1000 mg/L monoelement strontium stock solution (certified reference material, manufactured by Merck KGAA Darmstadt, Germany). The calibration solutions were matched to the acid-content concentration and, where necessary (e.g., for bones), to the concentration of the main components. As the analysis was conducted in dilute solutions (e.g., 50–70 mg femur sample in 100 ml volume), strong matrix effects were not expected. Nevertheless, we regularly checked for matrix effects by spiking the samples and evaluating the recoveries, which consistently fell within the range of 90–110%.

### Decorporol MS measurement

Decorporol complexes were analyzed by a Waters Acquity ultra-performance liquid chromatography instrument equipped with a Xevo-G2S qTOF mass spectrometer. The system was controlled by MassLynx 4.2 (Waters, Milford, MA, USA) software. Separations were performed by a Waters BEH Glycan column, 50 × 2.1 mm i.d., 1.7 μm particles, using a linear gradient of 80–20% acetonitrile (Buffer B) at 0.4 ml/min in 20 min, using 50 mM ammonium formate pH 4.4 as Buffer A. An amount of 1 µl (50 µg/ml) of sample was injected in all runs. The sample manager temperature was 15 °C, and the column temperature was 60 °C during each separation. During the MS analysis, 2.2 kV electrospray voltage applied to the capillary. The desolvation temperature was set to 120 °C, while the desolvation gas flow was 800 L/h. Mass spectra were acquired using positive ionization mode over the range of 100–1000 m/z. MS/MS fragments were obtained using 45 kV collision energy during the analysis.

### Statistical analysis

Data are expressed as the means of three independent experiments with biological replicates ± standard deviation (SD). In the case of in vitro data, statistical comparisons were conducted using Student’s t-test. P-values were considered statistically significant and marked with one star (*) if the *P*-value was less than 0.05, with two stars (**) if less than 0.01, and with three stars (***) if less than 0.001. For the in vivo experiments detailed in Fig. 5, statistical calculations were made by one-way ANOVA with Dunnett’s multiple comparisons, and the appropriate p values are indicated in the figure in each case. Sr incorporation was assessed by comparing all other treatment groups to the untreated controls receiving neither strontium nor Decorporol, and the resulting p values are shown in black. Decorporol treatment effect was assessed by a separate one-way ANOVA and post-hoc Dunnett’s test, since not all of the possible comparisons would be biologically meaningful. In this case, the 4x strontium and 4x Decorporol treatment group was compared only to the 4x strontium treated group and the untreated controls, and the results are shown in blue.

Statistics were calculated and graphs were compiled using GraphPad Prism 6.

## Electronic supplementary material

Below is the link to the electronic supplementary material.

Supplementary Material 1.

## Data Availability

The datasets used and/or analysed during the current study available from the corresponding author on reasonable request.
