# Supplementary material for: Development and validation of combined in vitro and in vivo assays for evaluating the efficacy of strontium-chelating compounds
Source: Sci Rep. 2025 Nov 28;15:43006. doi: 10.1038/s41598-025-06618-1 (PMC12672587; doi:10.1038/s41598-025-06618-1)
Supplement: Supplementary file 1 — Supplementary Material 1. [file 41598_2025_6618_MOESM1_ESM.pdf]

SUPPLEMENTARY MATERIAL

Development and Validation of Combined In Vitro and In Vivo Assays for Evaluating the Efficacy of Strontium-Chelating Compounds

Nóra Varga[1,2], Viola Pomozi[2], Eszter Kozák[2], Zsuzsa Erdei[1], Szilárd Tóth[2], Adriána Kutás[3], Zoltán Mucsi[3], Zsolt Rapi[4], Beatrix Kovács[2], Anett Matuscsák[5], Olivér Bánhídi[3], Béla Viskolcz[3], Csaba Váradi[3], Tamás Rácz[6], Katalin Német[1], Áron Szepesi[5]

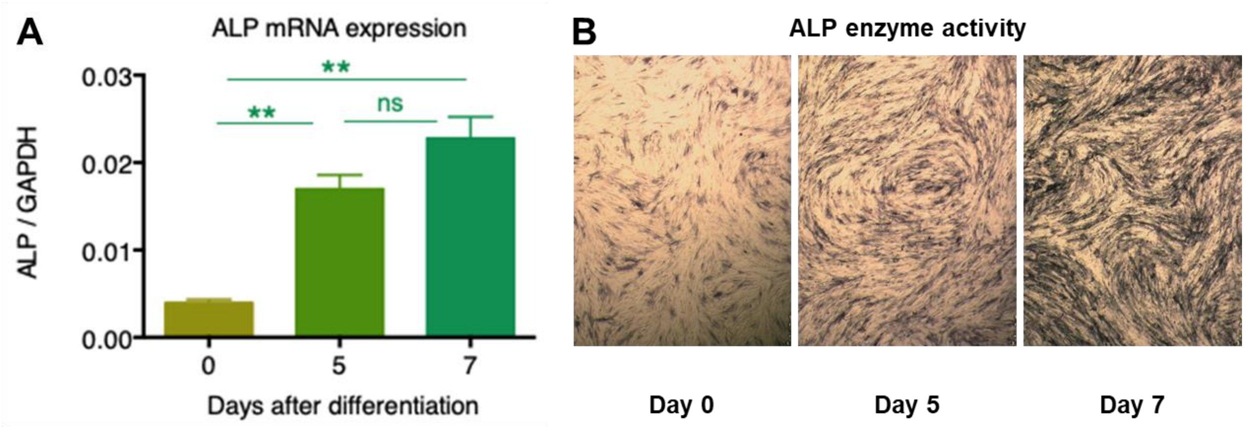

Supplementary Figure 1. *The ALP gene mRNA expression levels in the differentiated samples were determined using RT-qPCR measurements (A) and ALP enzyme activity measurements on days 0, 5, and 7 of osteogenic differentiation of bone marrow-derived MSCs (B).*

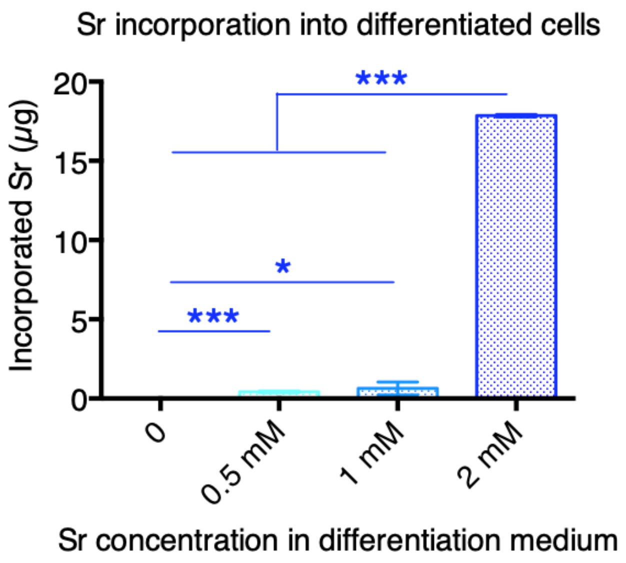

Supplementary Figure 2. *Strontium (Sr) incorporation was detected on the 10th day of osteogenic differentiation of primary bone marrow-derived MSCs by the ICP-AES technique. Cells were grown in differentiation medium supplemented with different concentrations of* $SrCl_2$*. The Y-axis represents the absolute amount of incorporated strontium into MSCs in micrograms [µg Sr].*

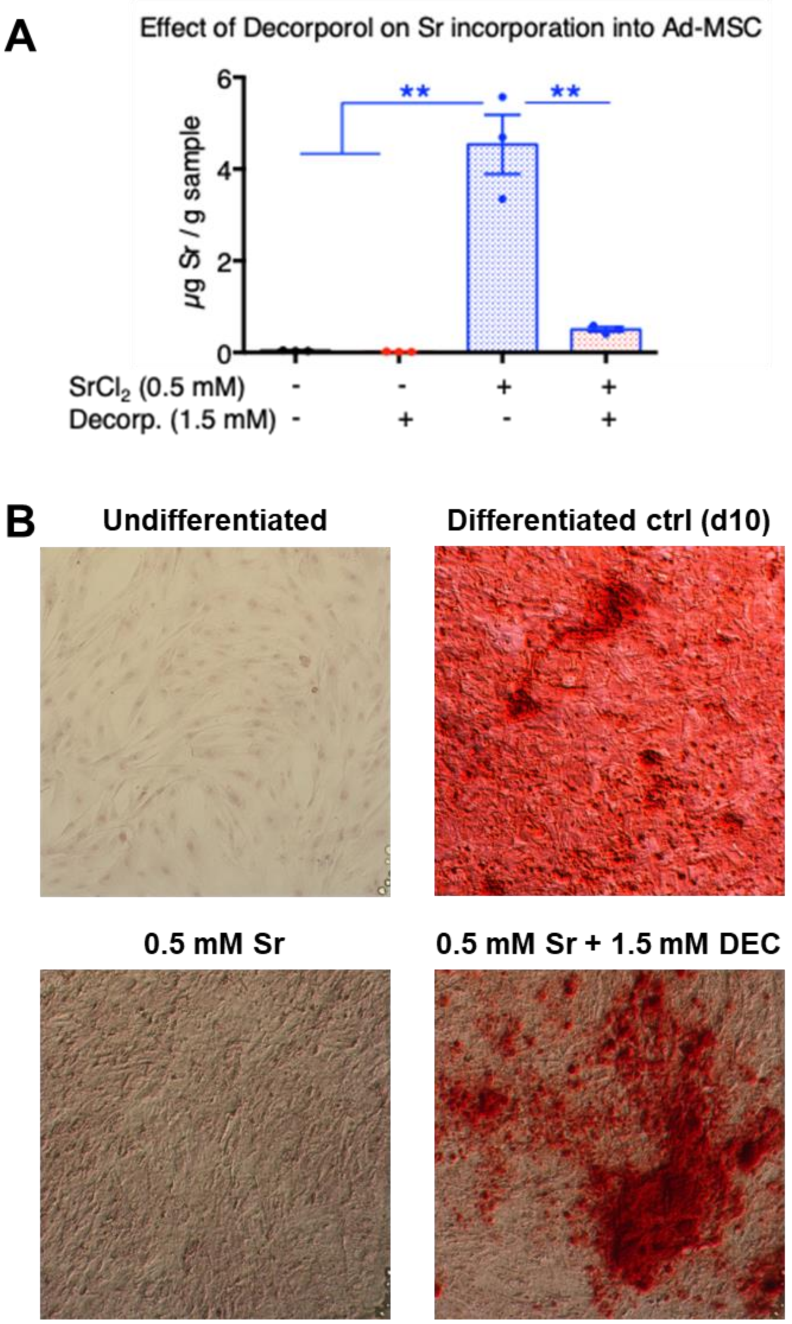

Supplementary Figure 3. *Detection of strontium (Sr) incorporation and the inhibitory effect on strontium deposition by ICP-AES technique on the 10th day of osteogenic differentiation of adipose-derived MSCs (Ad-MSCs) (A). Alizarin Red S staining of the Ad-MSC cells on the 10th day of osteogenic differentiation. Images captured at 4x magnification under a light microscope (B).*

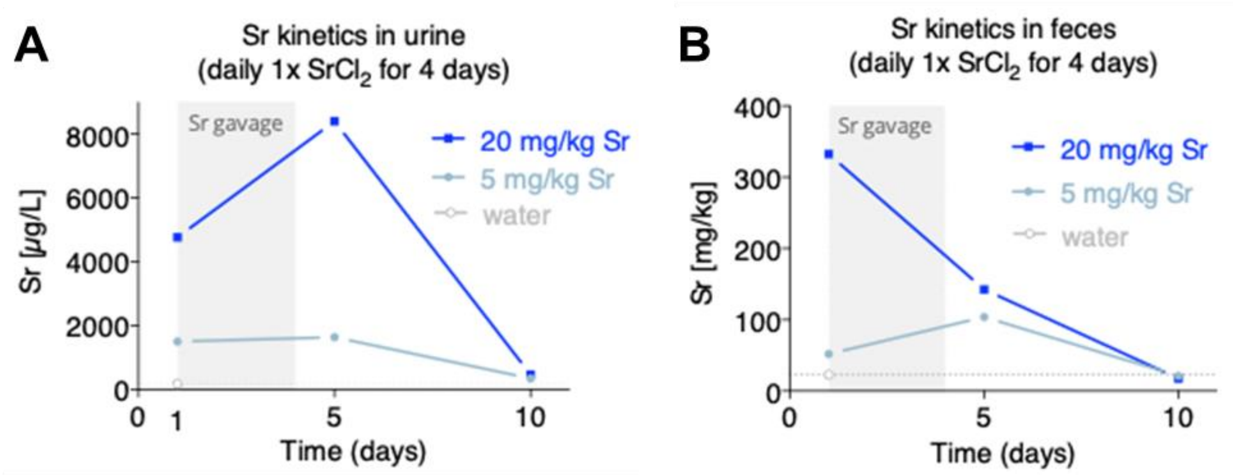

Supplementary Figure 4. Excretion kinetics of strontium in mice: *Strontium (Sr) was administered by oral gavage and detected using the ICP-AES technique. Strontium was cleared from the circulation and was excreted from the body into urine and feces (A, B).*
